# miR-4454 up-regulated by HPV16 E6/E7 promotes invasion and migration by targeting ABHD2/NUDT21 in cervical cancer

**DOI:** 10.1042/BSR20200796

**Published:** 2020-09-02

**Authors:** Hui Wang, Hui Hu, Zhenzhao Luo, Shuiyi Liu, Wangze Wu, Man Zhu, Jing Wang, Yingle Liu, Zhongxin Lu

**Affiliations:** 1 Department of Medical Laboratory, The Central Hospital of Wuhan, Tongji Medical College, Huazhong University of Science and Technology, Wuhan 430014, China; 2 Cancer Research Institute of Wuhan, The Central Hospital of Wuhan, Tongji Medical College, Huazhong University of Science and Technology, Wuhan 430014, China; 3 State Key Laboratory of Virology, College of Life Sciences, Wuhan University, Wuhan 430072, China

**Keywords:** α/β-hydrolase domain-containing 2, Cervical Cancer, HPV16 E6/E7, miR-4454, NUDT21

## Abstract

The abnormal expression of HPV16 E6/E7 activates oncogenes and/or inactivates tumor suppressor genes, resulting in the selective growth and malignant transformation of cancer cells. miR-4454 was selected by sequencing due to its abnormal high expression in HPV16 E6/E7 positive CaSki cell compared with HPV16 E6/E7 negative C33A cell. Overexpression of miR-4454 enhances cervical cancer cell invasion and migration. *ABHD2* and *NUDT21* are identified as a target gene of miR-4454.The effects of *ABHD2* and *NUDT21* on migration and invasion of CaSki and C33A cells were determined. The dual luciferase and RT-qPCR assays confirmed that miR-4454 might regulate its targets *ABHD2* and *NUDT21* to promote the proliferation, invasion and migration, whereas, inhibit the apoptosis in CaSki and C33A cells.

## Introduction

The incidence of cervical cancer ranks the second among all female tumors with nearly 500,000 women worldwide diagnosed with cervical cancer every year, nearly half of whom ultimately die of the disease [1,2]. It has been confirmed that cervical cancer is associated with high-risk human papillomavirus (HPV) infection, with HPV16 and HPV18 being the most common infectious agents found in 99.7% of cases of cervical squamous cell carcinoma and 94–100% of cases of cervical adenocarcinoma [1,3–6]. China’s morbidity and mortality associated with cervical cancer account for nearly one-quarter of the world’s total, representing a serious threat to women’s health [1].

In HPV-positive cervical cancers, all of the malignant cells contain at least one copy of the viral genome with transcriptional activity [7,8]. Recent studies have shown that non-coding microRNAs (miRNAs) are also regulated by E6 and E7 at the transcriptional and post-transcriptional level [6,9–13].

In the present study, we investigated the molecular mechanism of HPV16 E6/E7-regulating miRNA in cervical cancer progress. We used HPV16 E6/E7-positive human cervical cancer cell line CaSki as the major cell model. The aim of the present study was to elucidate the biological functions and potential target of miR-4454 in cervical cancer cells and provide evidence that miR-4454 may serve a potential candidate for the clinical treatment for cervical cancer.

## Materials and methods

### Cell culture

C33A cell [American Type Culture Collection (ATCC), Rockville, MD, U.S.A.], which is an HPV16 E6/E7-negative cell line, was maintained in 10 ml of 90% Eagle’s minimum essential medium (Gibco, Thermo Fisher Scientific, Inc., MA, U.S.A.) containing 10% fetal bovine serum (FBS) (Gibco, U.S.A.). The CaSki cell (ATCC, Rockville, MD, U.S.A.), which is an HPV16 E6/E7-positive cell line, was maintained in 10 ml of 90% RPMI-1640 medium (Gibco, U.S.A.) containing 10% FBS (Gibco, U.S.A.). After mixing, 1 ml of the medium was added to each well of the culture plate and placed in a 5% CO_2_, 37°C incubator.

### The differentially expressed gene was selected by sequencing

siRNA sequences are synthesized by Sangon Biotech (Shanghai) Co., Ltd. sh-E6: F:5´-GGAGCGACCCAGAAAGUUATT-3´,R:5´-UAACUUUCUGGGUCGCUCCTT-3´. Then, the interfering shRNA was screened and transfected into CaSki cell. Finally, CaSki cell and siRNA cell were sent to the personalbio (Shanghai, China) for sequencing and screening for differential miRNA. Sample processing: after total RNA was extracted, the RNA quality was detected. Then, the small RNA library was constructed by IlluminatheTruSeq Small RNA Sample prep Kit. The enriched library was amplified by PCR and purified by gel electrophoresis. The library quality was checked by Agilent High Sensitivity DNA Kit using Agilent 2100 Bioanalyzer. After testing the qualified library, the library was quantified by Quant-iT Picogreen dsDNA Assay Kit. Finally, single-stranded library was used as template to bridge PCR amplification, and primers were annealed and sequenced. Data analysis: the original data are de-joined and filtered, and the filtered sequence is de-reprocessed. The deleted small RNA sequence is annotated on the basis of the reference genome and the corresponding annotation abundance is given. After statistical analysis, the differentially expressed miRNAs were clustered and predicted, and the predicted target genes were enriched and analyzed.

### Construction of miR-4454 mimics and miR-4454 inhibitor

The sequence of the hsa-miR-4454 mature body is 5′-GGAUCCGAGUCACGGCA CCA-3′ (http://www.mirbase.org/cgi-bin/mature.pl?mature_acc=MIMAT0018976), which was synthesized by GenScript (Nanjing) Co., Ltd. (Nanjing, Jiangsu, China). The miR-4454 mimics/inhibitor (RIBOBIO, Guangzhou, China) was then successfully constructed based on this sequence.

### Construction of pre-ABHD2/NUDT21 and sh-ABHD2/NUDT21 plasmids

The template strand sequences were as follows: ABHD2 positive-sense 5′-GATCGCCAATGGGAGCGTAACAAGTTTCAAGAGAACTTGTTACGCTCCCATTGGCTTTTTT-3′, antisense:5′-AATTAAAAAAGCCAATGGGAGCGTAACAAGTTCTCTTGAAACTTGTTACGCTCCCATTGGC-3′; NUDT21positive-sense:5′-GATCGCGCATGAGGGAAGAATTTGATTCAAGAGATCAAATTCTTCCCTCATGCGCTTTTTT-3′, antisense:5′-AATTAAAAAAGCGCATGAGGGAAGAATTTGATCTCTTGAATCAAATTCTTCCCTCATGCGC-3′. The small hairpin RNA (shRNA) sequences were then obtained by polymerase chain reaction (PCR) amplification, and inserted in the vector plasmid pSIREN connected by EcoRI and BamHI enzymes (Thermo Fisher, MA, U.S.A.). Finally, the recombinant plasmids pSIREN-shRNA-ABHD2 and pSIREN-shRNA-NUDT21 were amplified and infected into the CaSki and C33A cells.The template strand sequence of ABHD2 was as follows: forward primer 5′-CGCGGATCCATGAATGCCATGCTG-3′, reverse primer 5′-CCGCTCGAGTCACT CCAGGTCGGCCT-3′. The vector plasmid pcDNA3.1 was digested and connected by EcoRI and XhoI enzymes (Thermo Fisher, MA, U.S.A.). The recombinant plasmids pcDNA3.1-pre-ABHD2 and pcDNA3.1-pre-NUDT21 were amplified and infected into the CaSki and C33A cells. The template strand sequence of ABHD2 was as follows: forward primer 5′-CGCGGATCCATGTCTGTGGTAC CG-3′, reverse primer 5′-CCGCTCGAGTCAGTTGTAAATAAAATTG-3′.

### Western blot

Total protein was extracted by RIPA buffer (Beyotime, Shanghai, China) involved in protease inhibitors. The protein concentration measured by BCA protein assay kit (Thermo Fisher Scientific). The supernatant soluble lysate was mixed with loading buffer and the solution boiled for 10 min and stored at −20°C. Isolated proteins were electrophoresed on 8% SDS-PAGE electrophoresisand transferred to a 0.22-μm PVDF membrane and blocked with 5% nonfat milk. Afterward, the membranes were then incubated with primary antibodies against GAPDH (1:5000, ab181602, Abcam, Shanghai, China), ABHD2 (1:1000, ab230417, Abcam, Shanghai, China), NUDT21 (1:3000, ab183660, Abcam, Shanghai, China), Caspase-3 (1:1000, ab13847, Abcam, Shanghai, China) and Cleaved Caspase-3 (1:1000, ab49822, Abcam, Shanghai, China) overnight at 4°C. The membranes were then incubated with the HRP-conjugated secondary antibody (1:10,000, Santa Cruz) and the protein level detected by chemiluminescence.

### Transwell assay

A transwell assay was performed to investigate the migration and invasion of C33A and CaSki cells. The cells were harvested for 24 h before cell suspension, and then the cells were digested to be used as signal cells at a 5 × 10^5^/ml concentration. The cells were suspended using a serum-free medium containing bovine serum albumin, and 100 µl of the cell suspension was added to the transwell chamber. The 24-well plate chamber was filled with 600 µl of medium per well containing 10% FBS, and then the plate was incubated for 24 h at 37°C. After incubation, tweezers were used to carefully remove the chamber. The upper chamber fluid was drained and approximately 800 µl of methanol was added to each well for fixation for 30 min at room temperature. The chamber was taken out and dyed with 1 ml of 0.5% Crystal Violet solution for 30 min. The cells were observed using a microscope (TS100-F, Nikon, Tokyo, Japan). The experimental protocol for the invasion assay of C33A and CaSki cells was similar to that of the migration assay, except for the addition of Matrigel (BD, NJ, U.S.A.) and the serum-free medium diluted at a 1:8 ratio at 4°C. The plate was coated with the upper chamber surface of the bottom membrane in the transwell membrane. The Matrigel was polymerized to a gel at 37°C for 30 min.

### 3-(4,5-dimethylthiazol-2-yl)-2,5-diphenyltetrazolium (MTT) assay

The logarithmic-phase cells from cell subculture were plated in 96-well plates with 180 µl (5 × 10^3^ cells) per well, and the plates were incubated at 37°C overnight. The effect of miR-4454, *ABHD2*, and *NUDT21* on the proliferation of HPV16-positive CaSki cells and HPV16-negative C33A cells was detected at 24, 48 and 72 h. After the addition of 20 µl MTT (Beyotime, Shanghai, China) solution (5 mg/ml) to the cell culture plates of different groups, the plates were incubated for 4 h. After the supernatant was removed, 150 μl of dimethyl sulfoxide (Beyotime) solution was added to each well and mixed, and the absorbance value of each well was measured at 490 nm.

### Flow cytometry assay

After the C33A and CaSki cells of the different treatment groups were collected, they were centrifuged at 1000 ×***g*** for 5 min, and then suspended by phosphate-buffered saline (PBS) and counted. Approximately 2 × 10^6^ cells/ml were suspended with binding buffer, and then centrifuged at 300 × *g* for 10 min [14]. Subsequently, 5 µl of Annexin V-FITC (Solarbio, Beijing, China) was added to 100 µl of the cell suspension and mixed at room temperature for 10 min, followed by the addition of 5 µl propidium iodide and mixed at room temperature for 5 min. Finally, the PBS was added to reach a constant volume (500 µl), and cell apoptosis was detected by flow cytometry within 1 h.

### Reverse transcription-quantitative PCR (RT-qPCR) assay

CaSki and C33A cells were harvested and the total RNA was extracted using Trizol reagent (Ambion, TX, U.S.A.). The total RNA was reverse-transcribed to cDNA by PrimeScript™ RT Master Mix (TaKaRa, Dalian, China) according to the manufacturer’s instructions. The mRNA was quantified by SYBR® Premix Ex Taq™ II kit (TaKaRa, Dalian, China). The following primers were designed by Nanjing Genscript Biological Technology Co., Ltd.: miR-4454F: 5′-GGGGGATCCGAGT CA-3′, R:5′-AACTGGTGTCGTGGAGTCGGC-3′; ABHD2F:5′-CGTTGACTACGCCCAGAA-3′, R:5′-AAGCCCACGACGACCAG-3′; NUDT21F:5′-AAACTACCTGGTGGTGAACT-3′, R:5′-CTTAGGCTTTGTAATATGTGC-3′; U6F:5′-CTCGCTTCGGCAGCACA-3′, R:5′-AACGCTTCACGAATTTGCGT-3′; β-actin F: 5′-ACACTGTGCCCATCTACG-3′, R:5′-TGTCACGCACGATTTCC-3′. The experiment was executed independently in three biological replicates. General thermal cycling conditions for PCR: 95°C, 3 min; 95°C, 5 s; 56°C, 10 s; 72°C, 25 s; (39 cycles) 65°C, 5 s; 95°C, 50 s.

### Dual luciferase reporter gene assay

The 3′ untranslated regions (UTRs) of the *ABHD2* and *NUDT21* genes were cloned and amplified in 293 cells. Mutations of the 3′ UTR of the *ABHD2* and *NUDT21* genes were generated by the QuickChange Site-Directed Mutagenesis kit (Stratagene, CA, U.S.A.) at the putative binding site of miR-4454. The wild and mutant *ABHD2/NUDT21* genes were cloned into the pmirGLO-vector (Promega, WI, U.S.A.) and then co-transfected with pmirGLO-3′ UTR-ABHD2 (50 ng) or pmirGlO-3′ UTR-NUDT21 (50 ng) and miR-4454 (50 nM) using Lipofectamine® 2000 (Invitrogen, MA, U.S.A.). The reporter gene plasmid and transcription factor expression plasmid-co-transfected 293 cells were seeded in 24-well plates and incubated for 12 h. After transfection, cell lysates were prepared for 48 h using Passive Lysis Buffer (Promega, WI, U.S.A.). The dual luciferase activity was then examined using the Luciferase Assay System (Promega, WI, U.S.A.).

### Statistical analysis

All data were analyzed by the data analysis software SPSS19.0 (SPSS, IL, U.S.A.), and are presented as the mean ± standard deviation. Data were analyzed by one-way analysis of variance followed by Dunnett’s or Duncan’s test. A *P*-value less than 0.05 was considered statistically significant.

## Results

### miR-4454 promotes the invasion, migration, proliferation, and suppresses apoptosis of HPV16 E6/E7-positive CaSki cell

**Figure 1:**
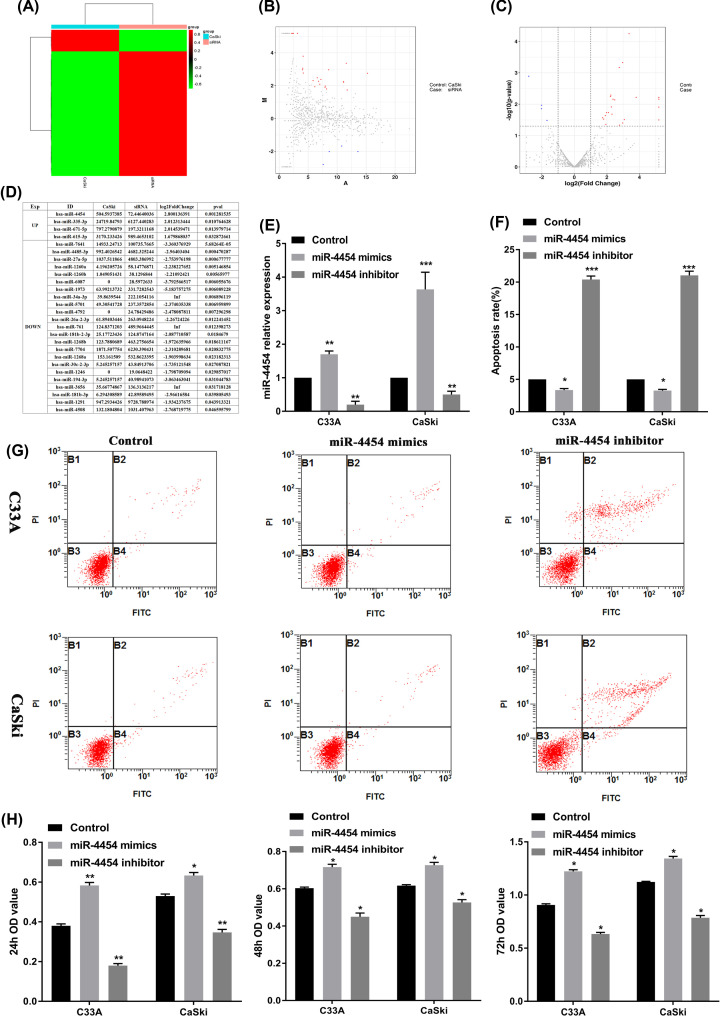
miR-4454 was highly expressed in HPV16 E6/E7 positive CaSki cell (**A**) The thermal map result for sequencing in CaSki cell. (**B** and **C**) The volcano map result for sequencing in CaSki cell. (**D**) The bioinformatics analysis for dramatically up- and down-regulated miRNAs in CaSki cell. (**E**) The expression of miR-4454 was detected by RT-qPCR in HPV16-positive cells (CaSki) and HPV16-negative cells (C33A) transfected with miR-4454 mimics and miR-4454 inhibitor. (**F**) Statistical analysis of the apoptosis rate in miR-4454 mimics- and miR-4454 inhibitor-transfected CaSki and C33A cells. (**G**) Flow cytometry images of cell apoptosis rates. (**H**) The proliferation of CaSki and C33A cells transfected with miR-4454 mimics and miR-4454 inhibitor at 24, 48, and 72 h. All data were obtained in triplicate; *, **, and *** indicate *P*<0.05, *P*<0.01, and *P*<0.001, respectively.

**Figure 2:**
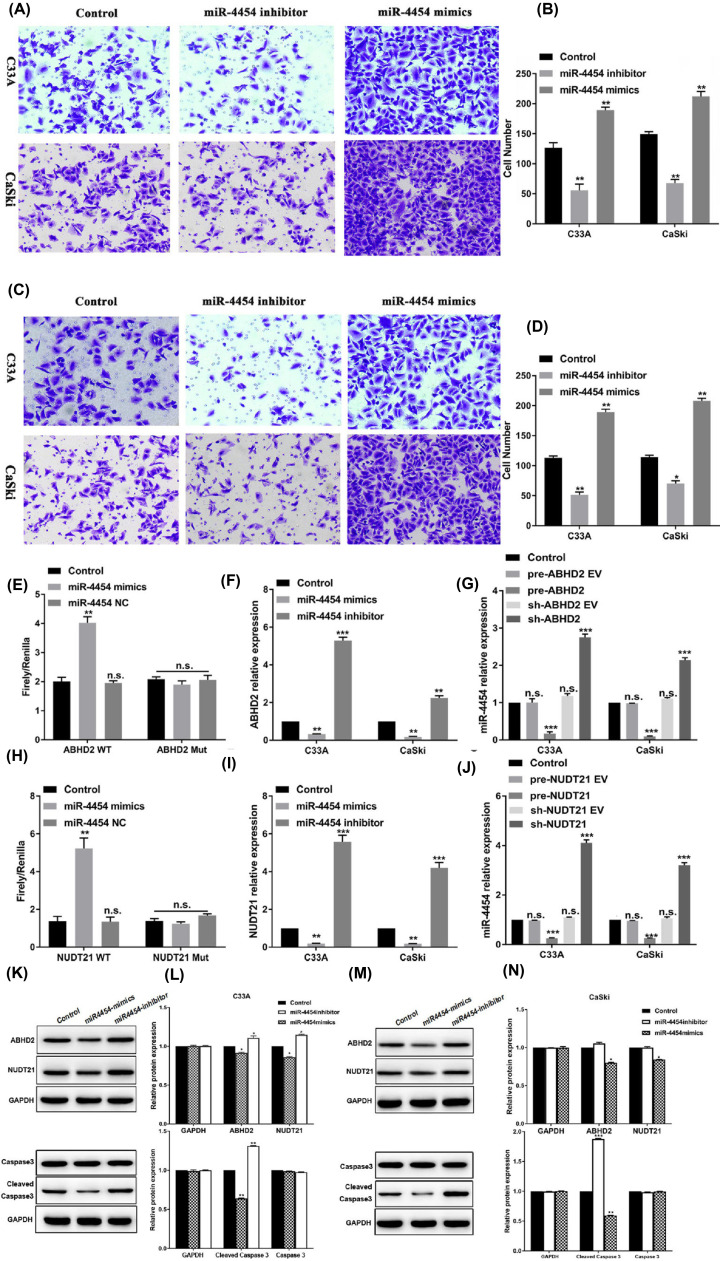
miR-4454 affected the invasion and migration on HPV16 E6/E7-positive CaSki cells (**A**) Transwell assay was used to detect cell invasion in CaSki and C33A cells. (**B**) Statistical analysis of the invasion of human cervical cancer cells (CaSki and C33A) treated with miR-4454 mimics and miR-4454 inhibitor. (**C**) Transwell assay was used to detect cell migration in CaSki and C33A cells. (**D**) Statistical analysis of migration in human cervical cancer cells (CaSki and C33A) treated with miR-4454 mimics and miR-4454 inhibitor. All data were obtained in triplicate; *, **, and *** indicate *P*<0.05, *P*<0.01, and *P*<0.001, respectively. (**E**) Interactions between miR-4454 and *ABHD2* evaluated in 293 cells by a dual luciferase assay. (**F**) Relative mRNA expression levels of *ABHD2* in cells transfected with the miR-4454 mimics and miR-4454 inhibitor. (**G**) Relative expression levels of miR-4454 in cells transfected with the pre-*ABHD2* and sh-*ABHD2.* (**H**) Interactions between miR-4454 and *NUDT21* evaluated in 293 cells by a dual luciferase assay. (**I**) Relative mRNA expression levels of *NUDT21* in cells transfected with the miR-4454 mimics and miR-4454 inhibitor. (**J**) Relative expression levels of miR-4454 in cells transfected with the pre-*NUDT21* and sh-*NUDT21.* (**K**) Western blot analyses of ABHD2, NUDT21, Caspase 3, and Cleaved Caspase 3 in C33A cells transfected with miR-4454 mimics and miR-4454 inhibitor. (**L**) Relative protein expression levels of ABHD2, NUDT21, Caspase 3, and Cleaved Caspase 3 in C33A cells transfected with the miR-4454 mimics and miR-4454 inhibitor. (**M**) Western blot analyses of ABHD2, NUDT21, Caspase 3, and Cleaved Caspase 3 in CaSki cells transfected with miR-4454 mimics and miR-4454 inhibitor. (**N**) Relative protein expression levels of ABHD2, NUDT21, Caspase 3, and Cleaved Caspase 3 in CaSki cells transfected with the miR-4454 mimics and miR-4454 inhibitor. Data were obtained in triplicate; *, **, and *** indicate *P*<0.05, *P*<0.01, and *P*<0.001, respectively.

The differentially expressed gene miR-4454 was selected by sequencing in HPV16 E6/E7 positive CaSki cell (Figure 1A–C). The sequencing result showed 23 underexpressed miRNAs (*P*<0.05) and 4 overexpressed miRNAs (*P*<0.05) in HPV16 E6/E7 positive cancer cell CaSki compared with HPV16 E6/E7 negative cancer cell. Among these 4 overexpressed, we found the largest fold change for miR-4454 (log2Fold Change = 2.8) (Figure 1D). In the present study, miR-4454 levels were confirmed by RT-qPCR (Figure 1E). The results showed that the HPV16 E6/E7 positive cell CaSki expressed higher levels of miR-4454 than HPV16 E6/E7 negative cell C33A. Then, CaSki and C33A cells were treated with the miR-4454 mimic/inhibitor to detect the effect on the proliferation, apoptosis, migration, and invasion. The results revealed that miR-4454 mimics inhibited the apoptosis of CaSki (*P*<0.05) and C33A (*P*<0.05) cells (Figure 1F,G), and promoted the proliferation of CaSki (*P*<0.001) and C33A (*P*<0.001) cells (Figure 1H). Although miR-4454 mimics significantly increased the proliferation of CaSki cells, there was no significant difference in the effect between CaSki and C33A cells. In addition, miR-4454 mimics significantly promoted the invasion (Figure 2A,B) (*P*<0.05) and migration (Figure 2C,D) (*P*<0.05) of both CaSki and C33A cells, and the effect was significantly greater in CaSki cells compared with C33A cells (*P*<0.05). These data suggested that miR-4454 can increase the invasion and migration of HPV16 E6, E7-positive cells.

### miR-4454 targets ABHD2 and NUDT21

The interactions between miR-4454 and *ABHD2/NUDT21* were evaluated in 293 cells by a dual luciferase assay (Figure 2E,H). In the *ABHD2* wild-type (WT) group, R/F was significantly increased in the miR-4454-transfected cells compared with the control (*P*<0.001) and miR-4454 negative control (NC)-transfected cells (*P*<0.01), while there was no significant difference in *ABHD2* mutated (Mut) group (*P*<0.001). Similarly, in the *NUDT21* WT group, F/R was significantly decreased in the miR-4454-transfected cells compared with the control (*P*<0.01) and miR-4454 NC-transfected cells (*P*<0.01), while there was no significant difference in *NUDT21* mutated (Mut) group (*P*<0.001). These results suggested that miR-4454 targets both *ABHD2* and *NUDT21*. To validate these results, an RT-qPCR assay and Western blot were used to detect the expression of *ABHD2 and NUDT21* mRNA levels and protein levels in CaSki and C33A cells (Figure 2F–I,K–N). Both of the targets were down-regulated in cells transfected with the miR-4454 mimics (*P*<0.05/*P*<0.01) and were up-regulated in cells transfected with the miR-4454 inhibitor (*P*<0.05) compared with the control. Whereas the cleaved Caspase 3 levels were decreased in miR-4454 overexepressed cells (*P*<0.05). Moreover, compared with C33A cells, the expression levels of *ABHD2* and *NUDT21* were markedly decreased in CaSki cells (*P*<0.05) (Figure 2G,J).

### ABHD2 and NUDT21 suppresses the invasion and migration of HPV16 E6/E7-positive CaSki cells

**Figure 3:**
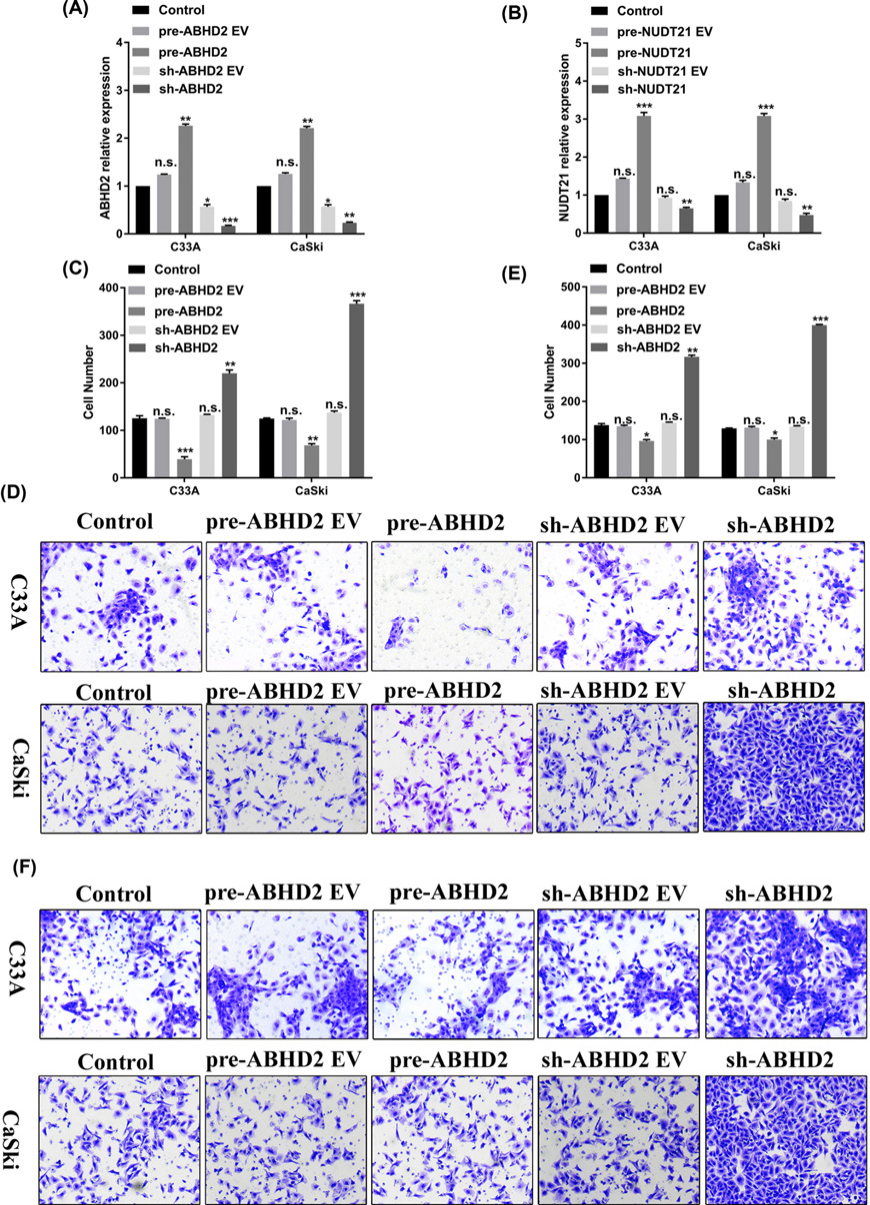
*ABHD2* affects the invasion and migration of HPV16 E6/E7-positive CaSki cells (**A**) Construction of pre-ABHD2 and sh-ABHD2 confirmed by RT-qPCR. (**B**) Construction of pre-NUDT21 and sh-NUDT21 confirmed by RT-qPCR. (**C**) Statistical analysis of invasion in human cervical cancer cells (CaSki and C33A) treated with pre-ABHD2 and sh-ABHD2. (**D**) Transwell pictures of cell invasion in CaSki and C33A cells. (**E**) Statistical analysis of migration in human cervical cancer cells (CaSki and C33A) treated with pre-ABHD2 and sh-ABHD2. (**F**) Transwell assay was used to detect cell migration in CaSki and C33A cells. All data were obtained in triplicate; *, **, and *** indicate *P*<0.05, *P<*0.01, and *P<*0.001, respectively.

**Figure 4:**
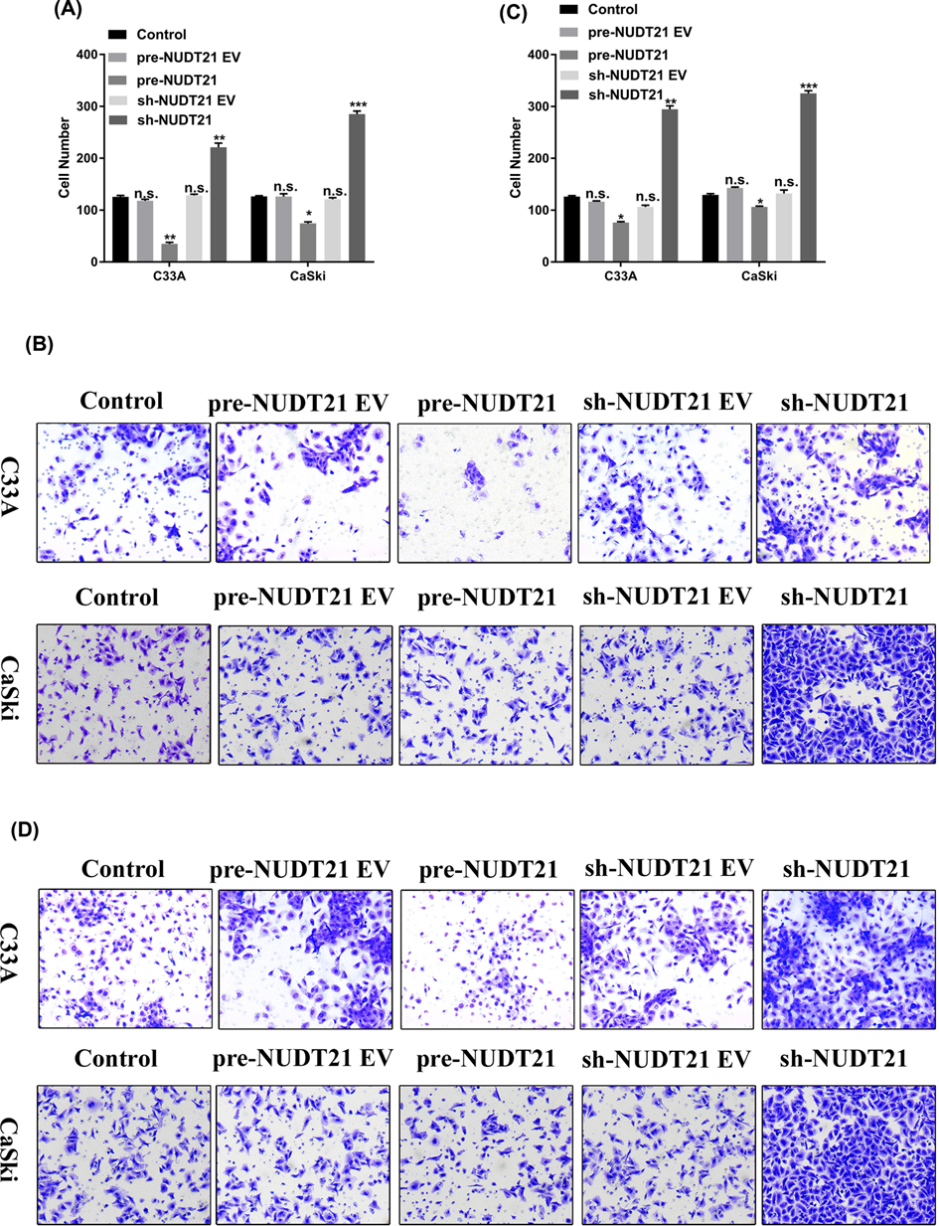
*NUDT21* affects the invasion and migration of HPV16 E6/E7-positive CaSki cells (**A**) Statistical analysis of invasion in human cervical cancer cells (CaSki and C33A) treated with pre-NUDT21 and sh-NUDT21. (**B**) Transwell assay was used to detect cell invasion in CaSki and C33A cells. (**C**) Statistical analysis of migration in human cervical cancer cells (CaSki and C33A) treated with pre- NUDT21 and sh-NUDT21. (**D**) Transwell assay was used to detect cell migration in CaSki and C33A cells. All data were obtained in triplicate; *, **, and *** indicate *P*<0.05, *P*<0.01, and *P*<0.001, respectively.

Since we demonstrated that *ABHD2* and *NUDT21* are targets of miR-4454, we next explored the effects of *ABHD2* and *NUDT21* on the invasion and migration of HPV16-positive cells (CaSki) and HPV16-negative cells (C33A). The RT-qPCR assay confirmed the successful construction of pre-ABHD2, sh-ABHD2, pre-NUDT21, and sh-NUDT21 plasmids. The expression of *ABHD2* was up-regulated in pre-ABHD2 group and was down-regulated in sh-ABHD2 group (Figure 3A). In addition, the expression of *NUDT21* was up-regulated in pre-NUDT21 group and down-regulated in sh-NUDT21 group (Figure 3B). The invasion and migration of the transfected cells were then detected by a transwell assay. Our results showed that pre-ABHD2 markedly decreased the invasion (Figure 3C,D) and migration (Figure 3E,F) of both C33A and CaSki cells (*P*<0.05), whereas sh-ABHD2 significantly increase the invasion and migration of C33A and CaSki cells (*P*<0.05 and *P*<0.01, respectively). These data indicated that *ABHD2* can decrease the invasion and migration of HPV16 cells (C33A and CaSki), or in other words, a lack of *ABHD2* can increase the invasion and migration of HPV16 cells (C33A and CaSki). Moreover, compared with C33A cells, the invasion and migration were promoted to a greater extent in CaSki cells (*P*<0.05). Similar results were obtained for pre-NUDT21 and sh-NUDT21 (Figure 4). Overall, these results suggest that HPV16 E6/E7 can affect cell invasion and migration via regulating miR-4454 targeting *ABHD2/NUDT21*.

### miR-4454 regulates invasion and migration in HPV16 cells through functional target ABHD2/NUDT21 *in vitro*

**Figure 5:**
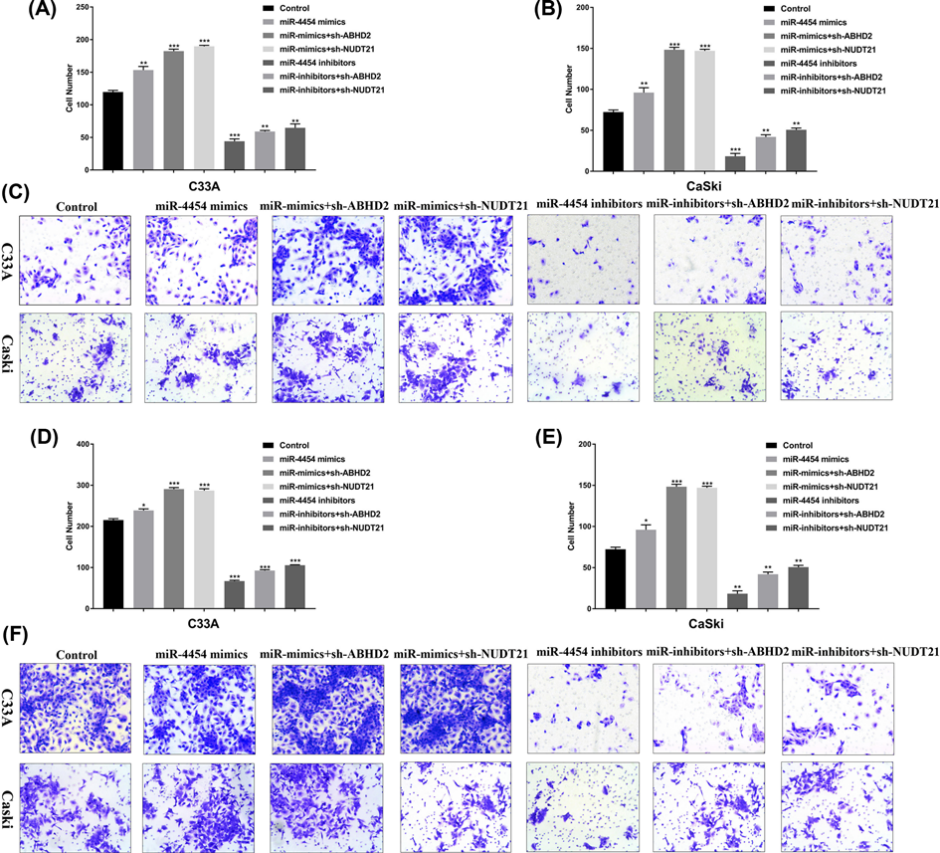
miR-4454 regulates invasion and migration in HPV16 cells through functional target *ABHD2/NUDT21* *in vitro* (**A**) Statistical analysis of invasion in human cervical cancer C33A cells treated with miR-4454 mimics, miR-4454 mimics with sh-ABHD2/NUDT21, miR-4454 inhibitors, and miR-4454 inhibitors with sh-ABHD2/NUDT21. (**B**) Statistical analysis of invasion in human cervical cancer CaSki cells treated with miR-4454 mimics, miR-4454 mimics with sh-ABHD2/NUDT21, miR-4454 inhibitors, and miR-4454 inhibitors with sh-ABHD2/NUDT21. (**C**) Transwell assay was used to detect cell invasion in C33A and CaSki cells. (**D**) Statistical analysis of migration in human cervical cancer C33A cells treated with miR-4454 mimics, miR-4454 mimics with sh-ABHD2/NUDT21, miR-4454 inhibitors, and miR-4454 inhibitors with sh-ABHD2/NUDT21. (**E**) Statistical analysis of migration in human cervical cancer CaSki cells treated with miR-4454 mimics, miR-4454 mimics with sh-ABHD2/NUDT21, miR-4454 inhibitors, and miR-4454 inhibitors with sh-ABHD2/NUDT21. (**F**) Transwell assay was used to detect cell migration in C33A and CaSki cells.

The invasion and migration of the transfected cells were detected by a transwell assay. Our results showed that transfected miR-4454 mimics and miR-4454 mimics with sh-ABHD2/NUDT21 markedly promote the invasion (Figure 5A–C) and migration (Figure 5D–F) in C33A cells and CaSki cells. Inversely, transfected miR-4454 inhibitors and miR-4454 inhibitors with sh-ABHD2/NUDT21 markedly decreased the invasion (Figure 5A–C) and migration (Figure 5D–F) in C33A cells and CaSki cells. Overall, these results suggest that miR-4454 regulates invasion and migration in HPV16 cells through functional target ABHD2/NUDT21 *in vitro*.

## Discussion

High-risk HPV16 E6/E7 can regulate the tumor suppressor p53 and pRb, respectively [15–17]. This activation consequently promotes the expression of c-Myc, and cell cycle protein A and E (CyclinA/E), which is beneficial to the synthesis of DNA and induces the progression of cells from G1 to S, thus promoting cell proliferation [17]. miR-4454 is involved in promoting the inflammation, catabolism, and cell death activity of chondrocytes in spinal osteoarthritis (facet joints) [18]. A previous study showed that miR-4454 was overexpressed in the bladder tumor, WBCs, and urine [19]. In tumor necrosis factor-alpha-stimulated human cervical cancer HeLa cells, miR-4454 was identified as an NF-κB-targeting miRNA, with roles in the anti-inflammatory, apoptosis, and other physiological processes of cervical cancer cells [19,20]. They suggested that miR-4454 may affect cancer cell migration, invasion, proliferation, and apoptosis. In the present study, we determined specific high expression of miR-4454 in HPV16 E6/E7 through a gene chip screening process. Therefore, we focused on the effects of miR-4454 and its downstream targets determined by querying the miRNAs target database on cervical cancer cells. Our data are in line with previous studies showing that miR-4454 is overexpressed in tumors, and is highly enriched in the HPV16 E6/E7 positive cervical cancer cell. Moreover, miR-4454 promoted the proliferation, invasion, and migration, and inhibited the apoptosis of CaSki cell compared with C33A cell.

The subsequent study was interested in the target of miR-4454 to further understand its effect on the progression of cervical cancer. *ABHD2* and *NUDT21* were selected as candidate targets of miR-4454 based on information from online databases. Yun et al. [21] described a novel mechanism by which the serine hydrolase *ABHD2* regulates calcium transfer from the endoplasmic reticulum to the mitochondria to affect cell death (anoikis) resistance, which is related to malignant phenotypes and is a hallmark of cancer. Yamanoi et al. [22] showed that down-regulation of *ABHD2* could promote a malignant phenotype and was associated with a poor prognosis for women with serous ovarian cancer. In addition, Obinata et al. [23] showed that the expression of *ABHD2* was up-regulated by androgen in LNCaP and VCaP cells. Disruption of *ABHD2* expression also promotes cell proliferation and migration of LNCaP cells. According to these results, *ABHD2* was proposed as a novel target for the diagnosis and treatment of prostate cancer.

In our study, we demonstrated that *ABHD2* is a target of carcinogenic miR-4454 and its expression is negatively correlated to that of miR-4454. Therefore, we supposed that suppression of *ABHD2* would have a carcinogenic effect. The invasion and migration of HPV16 E6/E7-positive CaSki cell were increased to a greater extent under *ABHD2* inhibition compared with the effects observed in HPV16-negative C33A cells, consistent with the previous study in prostate cancer [23]. The other study showed that knockout of *NUDT21* promoted the proliferation, metastasis, and tumorigenesis of HCC cells, whereas enhanced the expression of *NUDT21* had the opposite effect [24]. Our data also suggested that *NUDT21* is negatively correlated to miR-4454, and knockdown of *NUDT21* increased the invasion and migration of HPV16 E6/E7-positive CaSki cell compared with HPV16-negative C33A cell, indicating an effect in promoting the development of human cervical cancer.

In conclusion, miR-4454 was overexpressed in HPV16 positive cervical cancer cells, and HPV16 E6/E7 may affect cancer cell invasion and migration via miR-4454/*ABHD2/NUDT21* axis in cervical cancer cells. In follow-up experiments, we will further explore the specific roles of *ABHD2/NUDT21* in the development of HPV16-positive cervical cancer and its related mechanisms. This work is expected to provide new insights into the mechanism by which HPV16 E6/E7 regulate *ABHD2/NUDT21* through miR-4454 to better understand the malignant transformation of cervical cancer.

## Data Availability

The datasets used and/or analyzed during the current study are available from the corresponding author on reasonable request.

## Abbreviations

ABHD2: α/β-hydrolase domain-containing 2
ATCC: American Type Culture Collection
CyclinA/E: cell cycle protein A and E
FBS: fetal bovine serum
HPV: human papillomavirus
miRNA: microRNA
NUDT21: nudix nucleoside diphosphate linked moiety X-type, motif21
PCR: polymerase chain reaction
WT: wild-type
