# Correction: miR-4454 up-regulated by HPV16 E6/E7 promotes invasion and migration by targeting ABHD2/NUDT21 in cervical cancer

**DOI:** 10.1042/BSR-2020-0796_COR

**Published:** 2021-11-09

**Keywords:** α/β-hydrolase domain-containing 2, Cervical Cancer, HPV16 E6/E7, miR-4454

The authors of the original article “miR-4454 up-regulated by HPV16 E6/E7 promotes invasion and migration by targeting ABHD2/NUDT21 in cervical cancer” (*Biosci Rep* (2020) **40**(9); https://doi.org/10.1042/BSR20200796) would like to correct Figure 5. Due to their negligence, they had placed an incorrect image in Figure 5F (Caski; miR-mimics+sh-NUDT21). Figure 5F (Caski; miR-inhibitors+sh-ABHD2) had been duplicated in this panel in error. The correct figure is present in this Correction.

**Figure 5:**
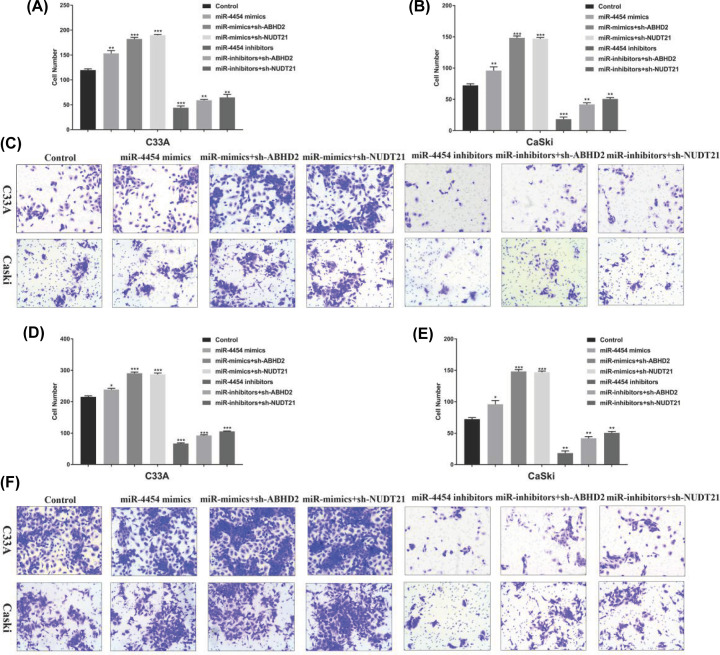
miR-4454 regulates invasion and migration in HPV16 cells through functional target *ABHD2/NUDT21in vitro* (**A**) Statistical analysis of invasion in human cervical cancer C33A cells treated with miR-4454 mimics, miR-4454 mimics with sh-ABHD2/NUDT21, miR-4454 inhibitors, and miR-4454 inhibitors with sh-ABHD2/NUDT21. (**B**) Statistical analysis of invasion in human cervical cancer CaSki cells treated with miR-4454 mimics, miR-4454 mimics with sh-ABHD2/NUDT21, miR-4454 inhibitors, and miR-4454 inhibitors with sh-ABHD2/NUDT21. (**C**) Transwell assay was used to detect cell invasion in C33A and CaSki cells. (**D**) Statistical analysis of migration in human cervical cancer C33A cells treated with miR-4454 mimics, miR-4454 mimics with sh-ABHD2/NUDT21, miR-4454 inhibitors, and miR-4454 inhibitors with sh-ABHD2/NUDT21. (**E**) Statistical analysis of migration in human cervical cancer CaSki cells treated with miR-4454 mimics, miR-4454 mimics with sh-ABHD2/NUDT21, miR-4454 inhibitors, and miR-4454 inhibitors with sh-ABHD2/NUDT21. (**F**) Transwell assay was used to detect cell migration in C33A and CaSki cells.

The authors apologise for the inconvenience caused by this mistake, and remain confident over the validity of the scientific conclusions and reproducibility of their original article.

